# Establishment of *TSH* β real-time monitoring system in mammalian photoperiodism

**DOI:** 10.1111/gtc.12063

**Published:** 2013-06-12

**Authors:** Kaori Tsujino, Ryohei Narumi, Koh-hei Masumoto, Etsuo A Susaki, Yuta Shinohara, Takaya Abe, Masayuki Iigo, Atsushi Wada, Mamoru Nagano, Yasufumi Shigeyoshi, Hiroki R Ueda

**Affiliations:** 1 Laboratory for Systems Biology, RIKEN Center for Developmental BiologyKobe, Hyogo 650-0047, Japan; 2 Graduate School of Science, Osaka University1-1 Machikaneyama, Toyonaka, Osaka, 560-0043, Japan; 3 Laboratory for Synthetic Biology, RIKEN Quantitative Biology Center2-2-3 Minatojima-minamimachi, Chuo-ku, Kobe, Hyogo, 650-0047, Japan; 4 Department of Anatomy and Neurobiology, Kinki University Faculty of Medicine377-2 Ohno-Higashi, Osakasayama City, Osaka, 589-8511, Japan; 5 Graduate School of Frontier Biosciences, Osaka UniversityYamadaoka 1-3, Suita, Osaka, 565-0871, Japan; 6 Laboratory for Animal Resources and Genetic Engineering, RIKEN Center for Developmental BiologyKobe, Hyogo 650-0047, Japan; 7 Department of Applied Biochemistry, Faculty of Agriculture, Utsunomiya University350 Mine-machi, Utsunomiya, Tochigi, 321-8505, Japan; 8 Center for Bioscience Research & Education (C-Bio), Utsunomiya University350 Mine-machi, Utsunomiya, Tochigi, 321-8505, Japan; 9 Utsunomiya University Center for Optical Research & Education (CORE)7-1-2 Yoto, Utsunomiya, Tochigi, 321-8585, Japan; 10 Department of Systems Pharmacology, Graduate School of Medicine, University of TokyoTokyo, 113-0033, Japan

## Abstract

Organisms have seasonal physiological changes in response to day length. Long-day stimulation induces thyroid-stimulating hormone beta subunit (*TSH*β) in the pars tuberalis (PT), which mediates photoperiodic reactions like day-length measurement and physiological adaptation. However, the mechanism of *TSH*β induction for day-length measurement is largely unknown. To screen candidate upstream molecules of *TSH*β, which convey light information to the PT, we generated *Luciferase* knock-in mice, which quantitatively report the dynamics of *TSH*β expression. We cultured brain slices containing the PT region from adult and neonatal mice and measured the bioluminescence activities from each slice over several days. A decrease in the bioluminescence activities was observed after melatonin treatment in adult and neonatal slices. These observations indicate that the experimental system possesses responsiveness of the *TSH*β expression to melatonin. Thus, we concluded that our experimental system monitors *TSH*β expression dynamics in response to external stimuli.

## Introduction

Many organisms adapt their physiological functions and behaviors to seasonal environmental changes by measuring day length, a biological process known as photoperiodism (Dawson *et al*. 2001; Ebling & Barrett 2008; Revel *et al*. 2009). Integration of external light information and endogenous time mediated by circadian clock genes (clockwork genes) is important for the photoperiodic responses (Hamner 1963; Pittendrigh & Minis 1964).

The pars tuberalis (PT) of the pituitary gland may be responsible for the photoperiodic responses because of the high expression levels of the clockwork genes and melatonin receptor 1a (MT1) in the PT (Morgan & Williams 1996; Lincoln *et al*. 2003; Ikegami & Yoshimura 2012). In mammals, it is suggested that nocturnal melatonin conveys darkness information to the PT, but the molecules conveying light information remain unknown (Hoffman & Reiter 1965; Morgan & Williams 1996). In a recent study, *thyroid-stimulating hormone beta subunit* (*TSH*β) gene was found to be rapidly induced by long-day stimulation in the PT and the product of *TSH*β, thyroid-stimulating hormone (TSH), has a functional role in seasonal testicular growth in birds (Yoshimura *et al*. 2003; Nakao *et al*. 2008). This *TSH*β induction is conserved even in mammals including melatonin-proficient mice (Hanon *et al*. 2008, 2010; Ono *et al*. 2008; Dardente *et al*. 2010; Dupre *et al*. 2010; Masumoto *et al*. 2010; Yasuo *et al*. 2010). Therefore, *TSH*β is a key factor for day-length detection and controls seasonal physiological changes.

Delivery of light information from the retina to the PT is an important pathway for inducing *TSH*β expression and regulating photoperiodic responses. Indeed, removal of the eyes, the suprachiasmatic nucleus or the pineal gland disrupts testicular responses in hamsters (Hoffman & Reiter 1965; Rusak & Morin 1976; Stetson & Watson-Whitmyre 1976). In the mouse PT, long-day stimulation induces a transcription cofactor *eyes absent 3* (*Eya3*) (Masumoto *et al*. 2010). This molecule regulates *TSH*β expression with DNA-binding transcription factor *sine oculis-related homeobox 1* (*Six1*) and several clockwork genes such as *thyrotroph embryonic factor* (*Tef*) and/or *hepatic leukemia factor* (*Hlf*) (Dardente *et al*. 2010; Masumoto *et al*. 2010). These molecular mechanisms have also been found in sheep (Dardente *et al*. 2010; Dupre *et al*. 2010). On the other hand, darkness information is mediated by melatonin that suppresses *Eya3* and *TSH*β expression in sheep and mice, respectively (Ono *et al*. 2008; Unfried *et al*. 2009; Dardente *et al*. 2010; Yasuo *et al*. 2010). Thus, the regulation of *Eya3* by external light conditions is critical to control *TSH*β expression for photoperiodism. However, the intracellular and extracellular signals that transfer the external light information to the PT to regulate *Eya3* and *TSH*β expression remain unknown.

In this study, we focused on how *TSH*β expression in the PT is regulated by long-day stimuli. To accomplish this, we established a real-time monitoring system for measuring *TSH*β expression dynamics for the purpose of screening candidate light information-transferring signals in the PT. We generated a genetic mouse model in which *TSH*β expression dynamics in the PT can be visualized by bioluminescence. Treatment of cultured PT slices with melatonin decreased bioluminescence activities, and these results showed that PT slices can response to melatonin in our culture condition. Thus, our mouse model could be used to search for molecules that relay external light signals in the PT.

## Results

### Generation of *TSH*β^*Luc*^ mice

**Figure 1:**
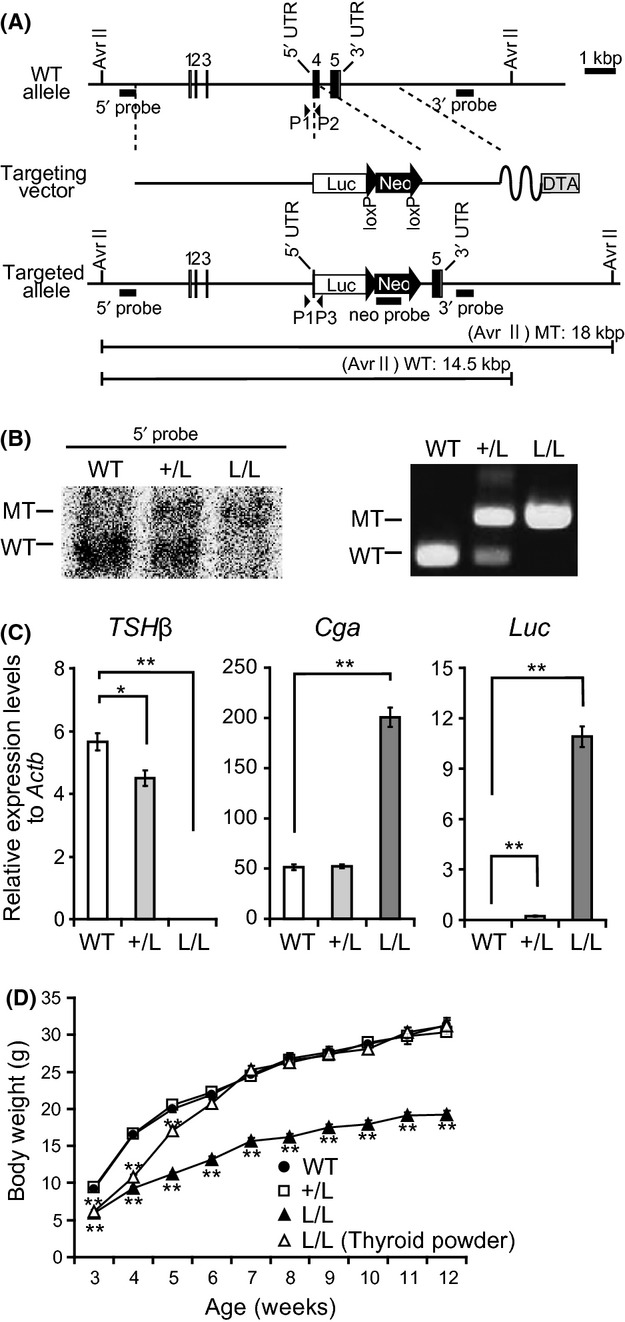
Generation of *TSH*β^*Luc*^ mice. (A) Targeting schema. Exons 1–5 are indicated by solid boxes. *TSH*β ORF is indicated by filled boxes. *Luc* ORF sequence was inserted in frame to the first ATG of exon 4 of the *TSH*β gene. The hybridization probes used for Southern blot analysis (5′, 3′ and Neo probes) and PCR primers for routine genotyping (P1-P3) are indicated by bold black lines and black arrowheads, respectively. (B) Southern blot analysis of the 5′ region (left) and genotyping PCR (right). Wild-type allele, heterozygous and homozygous mutant alleles are indicated as WT, +/L and L/L, respectively. One extra band was appeared in the +/L lane in genotyping PCR (right). (C) Expression levels of *TSH*β, *Cga* and *Luc* in the pituitary of *TSH*β^*Luc*^ mice by using quantitative RT-PCR (qPCR). The statistical differences between wild-type and heterozygous or homozygous TSHβ^Luc^ mice were calculated. *P < 0.05; **P < 0.01 (two-sample *t*-test, *n* = 6–8, average ± SEM). (D) Growth curves in male *TSH*β^*Luc*^ mice. The statistical differences between wild-type and heterozygous or homozygous *TSH*β^*Luc*^ mice were calculated. **P < 0.01 (two-sample *t*-test, *n* = 6–8, average ± SEM).

To monitor *TSH*β expression in the PT, we applied a knock-in approach in which 5′ exon of *TSH*β-coding sequence was knocked out and replaced with a firefly *Luciferase* (*Luc*) gene (Fig. 1A). In this scheme, the luc activity represents direct transcription from the endogenous *TSH*β promoter. Southern blot analysis confirmed that the desired mutation was successfully introduced (Fig. 1B, left), and genotypes of offspring were routinely analyzed by PCR (Fig. 1B, right). Animals were backcrossed and maintained on a CBA/N background because unlike the more common C57BL/6 strain, CBA/N mice maintain melatonin proficiency (Ebihara *et al*. 1986; Nakahara *et al*. 2003).

To verify the loss of *TSH*β transcript, we performed quantitative RT-PCR (qPCR) to measure the expression levels of *TSH*β and its related genes in the pituitary, where the highest *TSH*β expression level in the brain was observed (Kasukawa *et al*. 2011). By using primers aligned within exon 4, which was deleted by the targeting (Fig. 1A), *TSH*β mRNA was undetectable in homozygous *TSH*β^*Luc*^ mice. On the other hand, only 12% decrease in *TSH*β expression levels was observed in heterozygous *TSH*β^*Luc*^ mice compared with wild-type mice (Fig. 1C). The expression levels of *common glycoprotein* α-*subunit* (*Cga*), the alpha subunit of TSH, were not different between wild-type and heterozygous *TSH*β^*Luc*^ mice, but there was a four-fold increase in homozygous *TSH*β^*Luc*^ mice (Fig. 1C), suggesting antirepression of negative feedback loop due to the disruption of the TSH heterodimer. *Luc* expression levels were observed in heterozygous *TSH*β^*Luc*^ mice, significantly increased in homozygous *TSH*β^*Luc*^ mice and undetectable in wild-type mice, also suggesting the disruption of the TSH heterodimer (Fig. 1C).

One hundred and thirty-seven offspring from heterozygous matings were examined. 30 animals were wild-type (21%), 85 heterozygous (53%) and 35 animals homozygous *TSH*β^*Luc*^ mice (26%). These results are consistent with single-gene Mendelian inheritance, indicating that the *TSH*β null mutation did not lead to fetal loss and *TSH*β was not necessary for embryonic growth or viability. However, male and female homozygous *TSH*β^*Luc*^ mice were sterile. Weekly body weight results showed that homozygous *TSH*β^*Luc*^ mice display a low rate of growth, whereas heterozygous *TSH*β^*Luc*^ mice were indistinguishable from wild-type mice (Fig. 1D). These results indicate that slightly reduced *TSH*β expression and *Luc* expression do not affect the physiology in heterozygous *TSH*β^*Luc*^ mice. In addition, growth retardation in homozygous *TSH*β^*Luc*^ mice was recovered by supplementation with 100 ppm thyroid powder, consistent with the observation in TSH receptor (TSHR) knockout mice (Marians *et al*. 2002) (Fig. 1D). From these observations, we concluded that phenotypes of heterozygous *TSH*β^*Luc*^ mice are comparable with those of wild-type mice.

### Relationship between *TSH*β and *Luc* expression in *TSH*β^*Luc*^ mice

**Figure 2:**
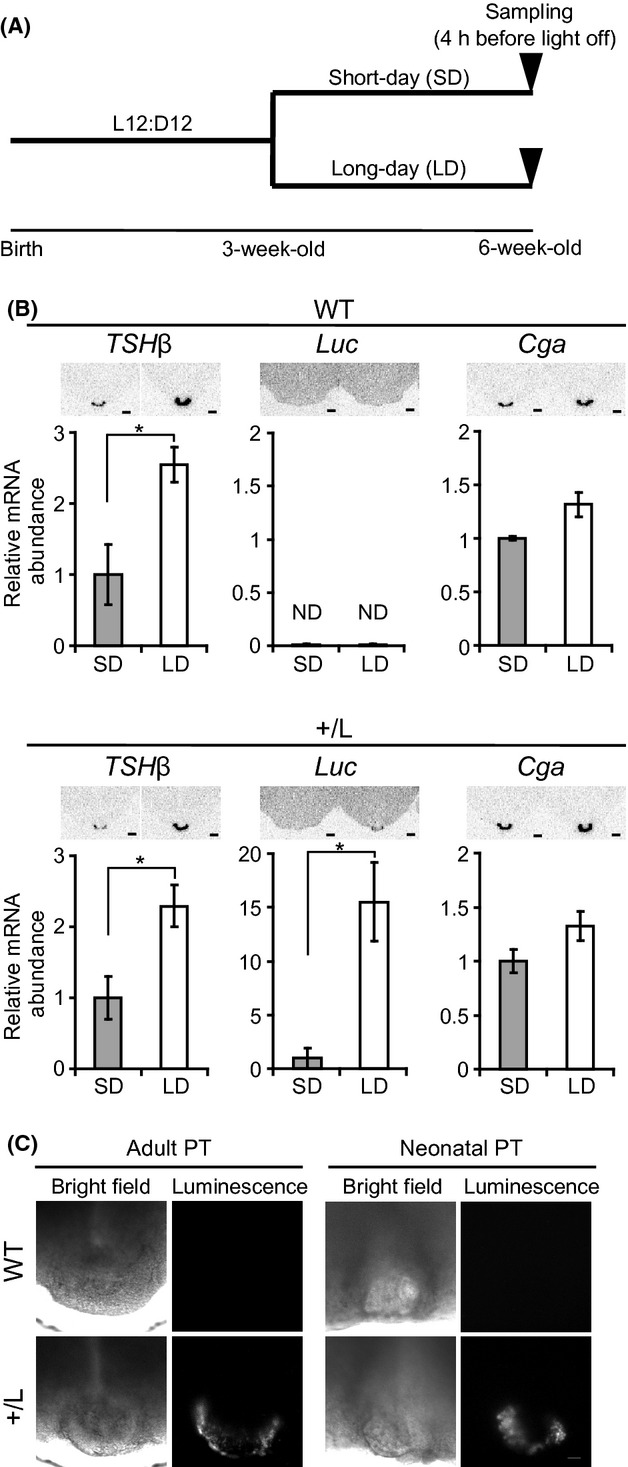
Photoperiodic gene responses in *TSH*β^*Luc*^ mice. (A) Experimental schema. 3-week-old mice were transferred to short-day (SD; light/dark = 8:16 hour) or long-day (LD; light/dark = 16:8 h) conditions and maintained for 3 weeks. Brain samples were collected 4 h before light-off (ZT4 of SD and ZT12 of LD). (B) Expressions of *TSH*β, *Luc* and *Cga* under SD and LD conditions in wild-type (WT) and heterozygous *TSH*β^*Luc*^ mice (+/L) measured by radioisotope (RI) *in situ* hybridization. The signal intensities were quantified and normalized by the values in SD samples except *Luc* measurement in WT mice. Scale bar: 300 μm. ND, not detected. *P < 0.05 (two-sample *t*-test, *n* = 3–5, average ± SEM). (C) Bioluminescence imaging of the PT. Adult PT slices were prepared from LD conditioned mice and neonatal PT slices were prepared from L12 : D12 conditioned mice. Bright-field and corresponding bioluminescence CCD images captured from the representative PT slices are shown. Scale bar: 100 μm (see also Fig. S1 and Video S1 in Supporting Information).

Next, we checked whether the knock-in *Luc* mice respond to changes in day length similar to endogenous photoperiodic genes in the PT. *TSH*β, *Luc* and *Cga* expressions in the PT of wild-type and heterozygous *TSH*β^*Luc*^ mice under different day-length conditions were analyzed by radioisotope (RI) *in situ* hybridization. After 3 weeks of exposure to short-day [light/dark = 8:16 hour (L8 : D16), zeitgeber time 0 (ZT0; ZT0 was defined as the time of light-on) = lights-on, ZT8 = lights-off] or long-day (L16 : D8, ZT0 = lights-on, ZT16 = lights-off) conditions, brain samples from each light condition were collected 4 h before light-off that corresponds to the same circadian phase under both conditions (Masumoto *et al*. 2010) (Fig. 2A). *TSH*β, *Luc* and *Cga* were detected only in the PT under both conditions, and the *TSH*β expression increased 2.5-fold under long-day conditions in wild-type and heterozygous *TSH*β^*Luc*^ mice. Although *Luc* expression was almost undetectable in heterozygous *TSH*β^*Luc*^ mice under short-day conditions and not detected in wild-type mice, its expression was clearly detected in heterozygous *TSH*β^*Luc*^ mice under long-day conditions (Fig. 2B). We also observed that *Cga* expression pattern was comparable in wild-type and heterozygous *TSH*β^*Luc*^ mice. These results indicate that the *Luc* expression represents the physiological photoperiodic responses of *TSH*β in the PT and does not affect the expressions of endogenous *TSH*β and *Cga*.

### PT-specific signals of the *TSH*β-Luc reporter

To detect bioluminescence derived from the *TSH*β promoter in the PT region, we prepared brain slices including regions from *TSH*β^*Luc*^ mice. First, we monitored the spatial pattern of the bioluminescence activity in the slice with a macrozoom microscope and a CCD camera. As expected, bioluminescence activity was observed only in the putative PT region surrounding the median eminence of the slices from 5- to 6-week-old (adult) heterozygous *TSH*β^*Luc*^ mice kept under long-day conditions for 2–3 weeks (Fig. 2C). These spatial bioluminescence patterns are compatible to signals of *Luc* mRNA (Fig. 2B,C). By contrast, no bioluminescence was observed in the slices from wild-type mice (Fig. 2C). Therefore, bioluminescence activity measured in the slice can be attributed to luc activity in the PT. In addition, similar bioluminescence pattern in the slices from adult mice was observed in the slices from postnatal 6- to 8-day-old (neonate) heterozygous *TSH*β^*Luc*^ mice kept under L12 : D12 (ZT0 = lights-on, ZT12 = lights-off) conditions (Fig. 2C). These observations are consistent with the *TSH*β expression *in vivo* (Schulze-Bonhage & Wittkowski 1990; Japon *et al*. 1994) and thus imply the possibility of slice usage from both adult and neonatal PT.

### Real-time bioluminescence monitoring of the *TSH*β-Luc reporter

**Figure 3:**
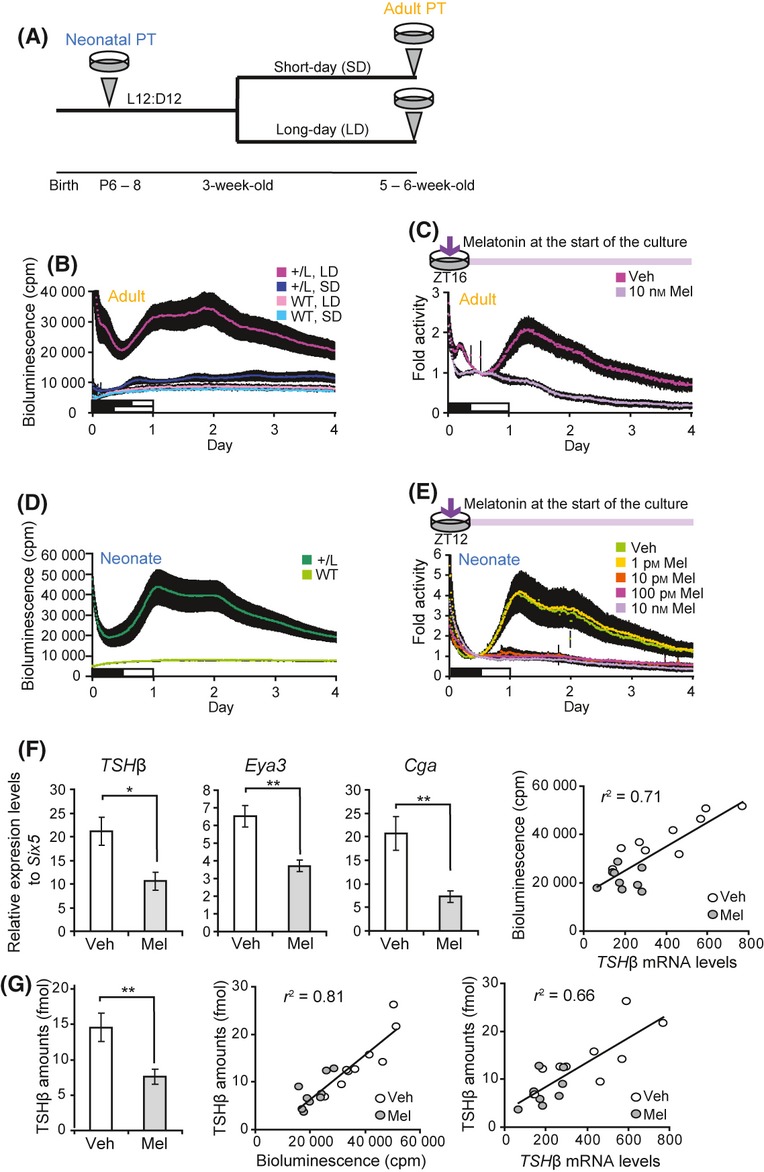
Real-time monitoring of the *TSH*β expression by bioluminescence. (A) Experimental schema. Neonatal PTs were collected at postnatal 6–8 (P6–8) under L12 : D12 conditions, and 5- to 6-week-old mice were collected after being maintained in short-day (SD) or long-day (LD) conditions for 2–3 weeks. (B and D) Real-time bioluminescence recording in adult or neonatal PT slices of indicated genotypes. White and black bars on the graphs indicate the subjective day and night on the following days after sampling, respectively [*n* = 2, wild-type (WT) mice or *n* = 6, heterozygous *TSH*β^*Luc*^ mice (+/L) in B; *n* = 5, WT and *n* = 17, +/L in D, average ± SEM]. (C and E) Real-time monitoring of the luciferase (luc) reporter activities in adult or neonatal PT slices after the addition of vehicle (Veh) or melatonin (Mel). The PT slices were prepared from animals kept under LD (adult, C) or L12 : D12 (neonate, E) conditions. Vehicle or melatonin was applied at the start of the culture. Normalized luc reporter activities of PT slices from +/L mice are shown (*n* = 4 in C and *n* = 3–4 in E, average ± SEM). (F) Confirmation of expression level changes in *TSH*β, *Eya3* and *Cga* in the cultured PT by qPCR analysis. Neonatal PT slices were treated with vehicle or 10 nm melatonin 48 h with PMT monitoring. Expression levels of *TSH*β, *Eya3* and *Cga* were normalized by the value of *Six5,* which was specifically expressed in the PT. *P < 0.05; **P < 0.01 (two-sample *t*-test, *n* = 9, average ± SEM). Correlation between *TSH*β mRNA levels and bioluminescence counts (right). White circles (Veh) and gray circles (Mel) indicate data points. The line is regression: *r*^2^ = 0.71. (G) Quantification of secreted amounts of TSHβ into the culture medium of the PT slices in 3F and S2 by mass spectrometry analysis. **P < 0.01 (two-sample *t*-test, *n* = 9, average ± SEM). Correlation between secreted TSHβ amounts (middle) and *TSH*β mRNA levels or bioluminescence counts (right). White circles (Veh) and gray circles (Mel) indicate data points. The line is regression: *r*^2^ = 0.81 (middle), *r*^2^ = 0.66 (right).

Next, we developed a real-time monitoring system for *TSH*β-Luc bioluminescence by a photomultiplier tube (PMT). Because Luc appears to be specifically expressed in the PT of the slice (Fig. 2C), photon counts by PMT measurements represent a mean bioluminescence of the tissue. First, the bioluminescence signals of slices from adult heterozygous *TSH*β^*Luc*^ mice kept under short- or long-day conditions for 2–3 weeks (adult short- or long-day slices) were measured (Fig. 3A,B). Significantly higher bioluminescence signals were detected from adult long-day slices than from adult short-day slices and wild-type slices. We further checked whether the slices have *in vivo*-like responsiveness to photoperiodic signals. We treated adult long-day slices with 10 nm melatonin from subjective ZT16 (ZT16 is the start of the culture and subjective night) (Fig. 3C). In agreement with previous *in vivo* studies (Ono *et al*. 2008; Unfried *et al*. 2009; Yasuo *et al*. 2010), a decrease in luc activity was observed by melatonin treatment. These results suggest that the experimental system keeps *TSH*β response to melatonin even in our culture condition.

One important application of this real-time monitoring system is to screen molecules that convey light signals under long-day conditions and induce the *TSH*β expression. Thus, we examined the possibility of using of neonatal tissues instead of adult tissues because of some advantages: (i) higher viability of tissues, (ii) ease of slice preparation and (iii) significant signal levels even in L12 : D12 conditioned animals rather than long-day conditioned animals (Fig. 2C). To check whether neonatal tissues can be used for the monitoring assay, luc activity in the PT slices of neonatal heterozygous *TSH*β^*Luc*^ mice kept under L12 : D12 conditions (neonatal slices) was measured (Fig. 3A). The robust bioluminescence activities of the neonatal slices were observed for over 2 days and gradually reached baseline levels after additional 2 or 3 days (Fig. 3D), with dynamics similar to those in adult long-day slices. Continuous luminescence in the PT for 2–4 days by imaging neonatal slices again confirmed that whole-tissue bioluminescence approximates activity in the PT (Fig. S1A and Video S1 in Supporting Information). Luminescence was also observed in individual PT cells over 4 days, but with noisy fluctuations (Fig. S1B and Video S1 in Supporting Information). Furthermore, the neonatal slices were treated with various concentrations of melatonin, and dose-dependent repression was observed (Fig. 3E). These patterns of bioluminescence with or without melatonin were similar to the results in the adult long-day slices and *in vivo*.

To independently confirm that the observed dynamics of bioluminescence is correlated with endogenous *TSH*β expressions in neonatal PT slices, the expression levels of *TSH*β, *Eya3* and *Cga*, which are known to be decreased by melatonin (Ono *et al*. 2008; Unfried *et al*. 2009; Dardente *et al*. 2010; Yasuo *et al*. 2010), were measured by qPCR. Cultured PT tissues with vehicle or 10 nm melatonin treatment for 48 h after the start of the culture were collected and subjected to qPCR analysis. The values of *sine oculis–related homeobox 5* (*Six5*) and *potassium voltage-gated channel, subfamily Q, member 5* (*Kcnq5*) were used for measuring the relative PT amounts of the samples and normalizing *TSH*β values and related genes, because *Six5* and *Kcnq5* expression is specific to the PT and does not respond to melatonin (Dupre *et al*. 2008; Masumoto *et al*. 2010). Consistent with *in vivo* observations (Ono *et al*. 2008; Unfried *et al*. 2009; Dardente *et al*. 2010; Yasuo *et al*. 2010) and bioluminescence results, the expression levels of endogenous *TSH*β, *Eya3* and *Cga* were significantly decreased under melatonin treatment, excluding the possibility that the decrease in the bioluminescence by melatonin might be an artifact (Fig. 3E,F and S2 in Supporting Information). In addition, there was a strong positive correlation between *TSH*β mRNA levels by qPCR analysis and bioluminescence counts of the PT slices by PMT measurements (*r*^*2*^ = 0.71; Fig. 3F, right). Furthermore, the culture medium of neonatal PT slices in Fig. 3F and S2 in Supporting Information was collected, and secreted TSHβ amounts were measured by mass spectrometry. In the melatonin-treated culture medium, secreted TSHβ amounts were 50% lower than vehicle-treated samples (Fig. 3G). There was a strong correlation between secreted TSHβ amounts and the bioluminescence counts (*r*^2^ = 0.81; Fig. 3G, middle) as well as the *TSH*β mRNA levels (*r*^2^ = 0.66; Fig. 3G, right). These results indicate that the responsiveness of the luc activity to an external signal reflects *TSH*β mRNA expression and secreted TSHβ protein.

Recently, it was reported that glutamine and glutamic acid induce the *TSH*β expression in the rat PT (Aizawa *et al*. 2012). To check whether the mouse PT also responds to glutamine and glutamic acid, the slices were treated with 1 mm glutamine or 1 mm glutamic acid after 2 h of glutamine and glutamic acid starvation. Although no response was observed by glutamine treatment, a transient increase in luc activity was observed by glutamic acid treatment. These results suggest that the slices can also respond to an inducer of *TSH*β (Fig. S3 in Supporting Information).

In summary, we visualized the *TSH*β expression dynamics in the PT by using adult and neonatal *TSH*β^*Luc*^ mice and observed responsiveness of the *TSH*β expression to an external signal.

## Discussion

In the present study, we established a *TSH*β real-time monitoring system in which the *TSH*β expression dynamics can be monitored as real-time bioluminescence activities. The difference in luc activity of PT slices derived from adult mice under short- or long-day conditions was consistent with *in vivo TSH*β expression levels in response to each day-length condition (Fig. 2 and 3B). Moreover, the luc activities of adult and neonatal PT slices were suppressed by melatonin treatment (Fig. 3C,E). We also observed dose-dependent suppression of *TSH*β in neonatal PT slices by melatonin (Fig. 3E) at physiological levels (Kennaway *et al*. 2002), which is consistent with the results using ovine PT cell cultures (Morgan *et al*. 1989). Furthermore, the luc activities were transiently up-regulated by glutamic acid treatment as seen in the rat PT (Aizawa *et al*. 2012) (Fig. S3 in Supporting Information). These observations suggest that PT slices possess at least *TSH*β responsiveness to melatonin and glutamic acid, and changes in *TSH*β expression dynamics under other stimuli are also expected to be observed by using our monitoring system.

Chronic melatonin treatment in this monitoring system may not necessarily the same as physiological conditions, comparing with nocturnal melatonin secretion *in vivo*. However, because *TSH*β was induced within several hours after long-day stimulation *in vivo*, this monitoring system is at least available for screening of candidate molecules upstream of *TSH*β, by observing luc activity during the same period. Transiently increased luc activity by glutamic acid treatment supports this idea (Fig. S3 in Supporting Information).

Several studies show that melatonin implantation (constant release of melatonin) has no effect on testicular regression in hamsters (Goldman *et al*. 1979). Furthermore, melatonin injection under long-day conditions does not suppress *TSH*β expression for at least 4 days in hamster (Yasuo *et al*. 2010), which is inconsistent with the immediate suppression presented in Fig. 3C. We speculate that difference between these *in vivo* observations and our *in vitro* results may be due to experimental conditions such as existence of *TSH*β-inducible signals. Therefore, careful design of experiments will be necessary in each purpose.

One possible application of our experimental system is to screen upstream light-information signals of the *TSH*β induction. Our system has several advantages for this purpose: (i) The PT slices can be used and easily treated with drugs compared with previous methods such as subcutaneous injection (Yasuo *et al*. 2007), intracerebroventricular injection (Ono *et al*. 2008) or dissociated PT cells (Morgan *et al*. 1989), (ii) availability of neonatal tissues increases the throughput of the experiment and viability of the slices. Previous reports support that neonatal PT has the similar photoperiodic properties to adult PT. First, high expression levels of *TSH*β (Schulze-Bonhage & Wittkowski 1990; Japon *et al*. 1994), MT1 receptor (Williams *et al*. 1991; Johnston *et al*. 2003) and rhythmic expression of the clockwork genes (Ansari *et al*. 2009) were observed in both embryonic and neonatal PT. Second, fetal and neonatal lambs showed changes in prolactin concentration in response to day length (Ebling *et al*. 1988, 1989).

We consider that neurotransmitters or humoral factors are possible candidates involving the extracellular upstream signals of *TSH*β. For example, there are several known neurotransmitters of deep brain photoreceptor–expressing cells in birds (Halford *et al*. 2009; Nakane *et al*. 2010). The afferent signals mediated by these neurotransmitters might transfer light stimuli to the PT. Additionally, receptors expressed in PT cells like Gs-coupled receptors and downstream cAMP signaling are possible candidates because these signals can antagonize decreases in *TSH*β by melatonin and the Gi-coupled MT1 receptor (Morgan *et al*. 1995).

Our system is also applicable to investigate the mechanism of photoinducible phase, by which the response of the PT to a light signal including *TSH*β induction is gated during the late night (Masumoto *et al*. 2010). Internal circadian time is probably involved in the determination of the gate timing, because photoperiodic responses can be induced by light stimuli at specific time (Ravault & Ortavant 1997). Our previous study showed that the brain slices of mPer2Luc mice containing PT exhibited circadian oscillations of luc (Masumoto *et al*. 2010). Also, it was reported that PER2::LUC rhythms in slices were detected in the median eminence/pars tuberalis (Guilding *et al*. 2009). These reports support that the PT region contains its intrinsic circadian oscillator. Analyzing circadian time dependency of *TSH*β expression after chemical/neurotransmitter stimulation in the PT may facilitate reconstitution of the *in vivo* phenomenon and help to show the details of gating.

The *TSH*β^*Luc*^ mice generated here are the first report of *TSH*β null mice. *TSH*β^*Luc*^ mice enabled us to study the photoperiodic roles of *TSH*β. Although *TSH*β is also expressed in the pituitary and functions as a part of the hypothalamus–pituitary–thyroid (HPT) axis, it is believed that the molecule in the PT acts differently from the HPT axis. This idea is supported by the results that *TSH*β expression in ovine PT did not change with thyroid-releasing hormone (TRH) and thyroid hormone (TH) treatment, because the lack of TRH and TH receptor or transcription factor *Pit-1* induces *TSH*β in the pituitary (Bockmann *et al*. 1997). Growth retardation observed in our homozygous *TSH*β^*Luc*^ mice (Fig. 1D) is consistent with previous observations in hypothyroid mice, such as TSHR knockout mice (Marians *et al*. 2002), the Snell dwarf mice (Cordier *et al*. 1976), the cog mice (Taylor & Rowe 1987) and the hyt mice (Beamer *et al*. 1981), and could be recovered by supplementation with thyroid powder (Fig. 1D). Thus, photoperiodic phenotypes in the *TSH*β^*Luc*^ mice, if any, can be discriminated from the effects of the impaired HPT axis by supplementation with thyroid powder. Because no obvious photoperiodic phenotype has not been identified in laboratory mice, such as testicular growth, which is a typical photoperiodic phenotype (Ono *et al*. 2008), it will be intriguing to further explore photoperiodic phenotypes in laboratory mice with reference to known seasonal physiological changes such as gonad growth in female European hamsters (Hanon *et al*. 2010), metabolic changes such as adipose depot mass and serum leptin (Atcha *et al*. 2000) and immune system such as NK cell cytolytic activity and phagocytosis in Siberian hamsters (Yellon *et al*. 1999; Martin *et al*. 2008). Even if no obvious photoperiodic phenotype can be identified in laboratory mice, molecular mechanisms underlying the induction of *TSH*β by long-day light exposure are evolutionary conserved (Hanon *et al*. 2008, 2010; Nakao *et al*. 2008; Ono *et al*. 2008; Dardente *et al*. 2010; Dupre *et al*. 2010; Masumoto *et al*. 2010; Yasuo *et al*. 2010). Therefore, *TSH*β^*Luc*^ mice, established in this study, will provide useful insights into the evolutionary conserved molecular mechanism of photoperiodism.

## Experimental procedures

### Generation of *TSH*β^*Luc*^ mice

*TSH*β^*Luc*^ mice [Tshb(Luc); Acc. No. CDB0713K: http://www.cdb.riken.jp/arg/mutant%20mice%20list.html] were generated as follows: a 1.9 kbp of Luc cassette that contains *Luciferase* (*Luc*)-coding region and simian virus 40 polyadenylation signal was amplified by PCR from pGL4.10 (Promega, Madison, WI, USA) using Luc-F-EcoRI (5′-AGAATTCTACTGTTGGTAAAGCCACCATGGAAGAT-3′) and Luc-R-NotI (5′-CGGAGCGGCCGCGATTTTACCACATTTGTAGAGGTTTTACTTGC-3′) primers (Hokkaido System Science, Sapporo, Japan). The amplified product was ligated into the EcoRI/NotI site of pBluescript SK (−) (Stratagene, La Jolla, CA, USA). The 6.3-kb 5′-arm was PCR-amplified from a bacterial artificial chromosome (BAC) clone containing *TSH*β (RP24-230F23; BACPAC Resources, Oakland, CA, USA) using 5-F-SalI (5′-GCAGGTCGACCAATAGAGGGAACAGAATAGTCCCAAACG-3′) and 5-R-BsmBI (5′-ACGGCGTCTCTCATGCTGAATCAGAGAGAAACATCAAAGAGCTC-3′) primers, and the amplicon was ligated into the SalI/NcoI site upstream of the *Luc* of pBluescript SK (−) to make the 5′ arm-Luc cassette, in which the Luc cassette was connected in frame to the first ATG of the *TSH*β gene. The 5′ arm-Luc cassette was inserted into the SalI/NotI site of the PGK-Neo-pA/DT-A vector (detailed descriptions of the vector are available from http://www.cdb.riken.go.jp/arg/cassette.html). The 2.8-kbp 3′-arm was also PCR-amplified from the BAC clone using 3-F-AvrII (5′-GACCCTAGGATGTTGTTCAATGCATTTCTTTTAGCTGTAA-3′) and 3-R-SalI (5′-ATTAGTCGACGTACCATGCTATGCTGTTAACCTGCAATAC-3′) primers, and the amplicon was ligated into the XbaI/XhoI site of the PGK-Neo-pA/DT-A vector. The resultant targeting vector was linearized with the AscI site and introduced into TT2 embryonic stem cells by electroporation (Yagi *et al*. 1993; Murata *et al*. 2004). Screening of homologous recombinant embryonic stem cells and production of chimera mice are described elsewhere (http://www.cdb.riken.jp/arg/Methods.html). PCR primers used to routinely identify the wild-type allele are P1 (5′-CGCAGGGCCCAGGGATAAGTAACCAGTCAG-3′) and P2 (5′-ACCCGTGTCATACAATACCCAGCACAGATGGTG-3′); those to identify the mutant allele are P1 (5′-CGCAGGGCCCAGGGATAAGTAACCAGTCAG-3′) and P3 (5′-ACAGCCACACCGATGAACAGGGCACCCAAC-3′) (Fig. 1A). PCR products of 305 and 446 bp were derived from wild-type and mutant alleles, respectively. For Southern blot analysis, genomic DNA extracted from ES cells or a mouse tail was digested with AvrII, and resulted bands were detected with radioactive probes (14.5- and 18-kbp bands from wild-type and mutant alleles, respectively, in Fig. 1A). Many laboratory mouse strains such as C57BL/6 lack melatonin production ability because of natural knockdown of the serotonin *N*-acetyltransferase (AANAT) activity (Roseboom *et al*. 1998) and low expression levels of hydroxyindole *O*-methyltransferase (HIOMT) protein (Kasahara *et al*. 2010; Shimomura *et al*. 2010), which are rate-limiting enzymes for melatonin synthesis. By contrast, CBA/N mice are melatonin proficient and they show significant *TSH*β induction after long-day stimulation (Ono *et al*. 2008; Masumoto *et al*. 2010). Thus, *TSH*β^*Luc*^ mice were backcrossed for more than 10 generations and maintained on a CBA/N background for all experiments except the measurement of growth in Fig. 1D (more than four generations).

### Animals

Mice were carefully kept and handled according to the RIKEN Regulations for Animal Experiments. For *in situ* hybridization experiments, L12 : D12 conditioned male 3-week-old wild-type and heterozygous *TSH*β^*Luc*^ mice were housed under short-day conditions (L8 : D16, 400 lux) or under long-day conditions (L16 : D8, 400 lux) for 3 weeks.

For neonatal PT slice culture experiments, neonatal wild-type and heterozygous *TSH*β^*Luc*^ mice were generated by overnight breeding of heterozygous *TSH*β^*Luc*^ and CBA/N mice (Japan SLC, Hamamatsu, Japan). Pregnant mice and pups were housed under L12 : D12 conditions (400 lux) because it was reported that exposure to different light/dark conditions from birth affects circadian properties (Ciarleglio *et al*. 2011). For adult PT slice culture experiments, L12 : D12 conditioned 3-week-old wild-type and heterozygous *TSH*β^*Luc*^ mice were housed under short- or long-day conditions for 2 to 3 weeks. For other experiments, *TSH*β^*Luc*^ mice were housed under L12 : D12 conditions. All mice were given commercial chow and water *ad libitum* except the supplementation of thyroid powder experiment. Homozygous *TSH*β^*Luc*^ mice were given commercial chow with 100 ppm thyroid powder (Sigma, St. Louis, MO, USA) and water *ad libitum* (Fig. 1D).

### *In situ* hybridization

Mice were deeply anesthetized with isoflurane (Mylan, Tokyo, Japan) and intracardially perfused with 10 mL saline and 20 mL of a fixative containing 4% paraformaldehyde in 0.1 m phosphate buffer (PB), pH 7.4. Mouse brain samples were post-fixed in the same fixative for 24 h at 4 °C, soaked in PB containing 20% sucrose for several days and stored frozen at −80 °C for further use. *In situ* hybridization was carried out as previously described (Shigeyoshi *et al*. 1997; Masumoto *et al*. 2010). Serial coronal sections (40 μm thick) of the mouse brain were prepared using a cryostat (CM 1850; Leica Microsystems, Wetzlar, Germany). To prepare probes, fragments of cDNA were obtained by PCR and subcloned into the pGEM-T easy vector (Promega). Radiolabeled probes were generated using ^35^S-UTP (PerkinElmer, Norwalk, CT, USA) via a standard protocol for cRNA synthesis. The primers used in the construction of *in situ* hybridization cRNA probes were as follows:

TSHβ cRNA probe:

Forward primer: 5′-TGGGTGGAGAAGAGTGAGCG-3′
Reverse primer: 5′-ACCAGATTGCACTGCTATTG-3′

Cga cRNA probe:

Forward primer: 5′-GCAGGCACTGAAAAATCCAGAGACATTGTTC-3′
Reverse primer: 5′-ACACACAGCGCCATTGAATGGCTC-3′

Luc cRNA probe:

Forward primer: 5′-CACCGGTAAGACACTGGGTGTGAACCAGC-3′
Reverse primer: 5′-CACGGCGATCTTGCCGCCCTTCTTGGC-3′

### PT slice culture

*TSH*β^*Luc*^ mice were decapitated 1–4 h before light-off. PT slices were prepared from neonatal pups (6–8 days old) or adult mice (5 to 6 weeks old). For neonatal PT slice culture experiments, brains were rapidly removed and were cut by a tissue chopper (McIlwain Tissue Chopper; McIlwain Laboratory Engineering, Gomshall, Surrey, UK) to a thickness of 350 μm. For adult PT slice culture experiments, brains were rapidly removed and were cut by a vibratome type linearslicer (PRO7; Dosaka EM, Kyoto, Japan) to a thickness of 300 μm. The PT region slices were dissected using a surgical knife. The slices were then placed on a culture membrane (MilliCell-CM; Millipore, Bedford, MA, USA) and set on a dish with 1.2 mL culture medium containing DMEM/F12 (Invitrogen, Carlsbad, CA, USA) supplemented with 300 mg/L NaHCO3 (Sigma), 20 mg/L kanamycin (Invitrogen), 100 mg/L apo-transferrin (Sigma), 100 μm putrescine (Sigma), 20 nm progesterone (Sigma), 30 nm sodium selenite (Sigma) and 1 mm luciferin (Biosynth, Staad, Switzerland). The dish was sealed with silicone grease (HVG; Dow Corning-Toray, Tokyo, Japan). Explants from different pups were cultured separately, and only those of appropriate genotypes were chosen for further experiments. Slices were treated with vehicle [ethanol (Sigma)] or melatonin (Sigma) (Fig. 3 and S2 in Supporting Information). For glutamine and glutamic acid starvation, culture medium with DMEM/F12 without glutamine and glutamic acid (Cell Science & Technology Institute, Sendai, Japan) was used. For glutamine or glutamic acid treatments, the glutamine- and glutamic acid–starved culture medium was replaced with the above fresh culture medium or with added glutamine (Sigma) or glutamic acid (Sigma) (Fig. S3 in Supporting Information).

### Quantitative RT-PCR (qPCR)

For qPCR experiments of the pituitary, *TSH*β^*Luc*^ mice were decapitated at ZT2 to 6 under L12 : D12 conditions to collect the part of the tissue embedded in the sella turcica (pituitary fossa) from 12-week-old male *TSH*β^*Luc*^ mice and temporarily stocked at −80 °C for further use. The total RNA was prepared from each pituitary, using RNeasy micro kit (Qiagen, Valencia, CA, USA). The cDNAs were synthesized from 0.25 μg of the total RNA using Superscript III transcriptase (Invitrogen). qPCR was performed using SYBR Green PCR Master Mix (Applied Biosystems, Foster City, CA, USA).

For qPCR experiments of the cultured PT samples, the cultured PT samples were collected 48 h after the start of the culture. The total RNA was prepared from each cultured PT, using NucleoSpin RNA XS kit (Takara, Otsu, Japan). The cDNAs were synthesized from 0.05 μg of the total RNA of the cultured PT using Superscript Vilo transcriptase (Invitrogen). qPCR was performed using Quantitect SYBR Green PCR mastermix (Qiagen).

qPCR was performed using the ABI PRISM 7900 (Applied Biosystems) at the following conditions: Samples contained 1 × SYBR Green PCR Master Mix or QuantiTect SYBR Green PCR Master Mix, 0.8 μm primers and 1/50 synthesized cDNA in a 10-μL volume. The PCR conditions were as follows: 10 min at 95 °C, then 45 cycles of 15 s at 94 °C, 1 min at 59 °C. The absolute cDNA abundance was calculated using a standard curve obtained from murine genomic DNAs. We used *TATA box-binding protein* (*Tbp*) or *actin, beta* (*Actb*) as the internal controls. Primer information is as follows:

*TSH*β mRNA (exon 4):

Forward primer: 5′-GTGGGCAAGCAGCATCCTTTTG-3′
Reverse primer: 5′-GCACACTCTCTCCTATCCACGTAC-3′

*Cga* mRNA:

Forward primer: 5′-TGCTGAGCCGAGCCATTCAATG-3′
Reverse primer: 5′-GAAGTCTGGTAGGGAGGAGGTGG-3′

*Luc* mRNA:

Forward primer: 5′-TCCTCAACGTGCAAAAGAAGCTACC-3′
Reverse primer: 5′-GTCGGTCTTGCTATCCATGATGATGATC-3′

*Actb* mRNA:

Forward primer: 5′-TTGTCCCCCCAACTTGATGT-3′
Reverse primer: 5′-CCTGGCTGCCTCAACACCT-3′

*TSH*β mRNA:

Forward primer: 5′-CTGCATACACGAGGCTGTCAG-3′
oReverse primer: 5′-CCCCAGATAGAAAGACTGCGG-3′

*Eya3* mRNA:

Forward primer: 5′-TTCACAGCTCCAAGTAGAATCTGACT-3′
Reverse primer: 5′-TATGGAAGCGCCATGAGCTT-3′

*Tbp* mRNA:

Forward primer: 5′-CCCCCTCTGCACTGAAATCA-3′
Reverse primer: 5′-GTAGCAGCACAGAGCAAGCAA-3′

### PMT bioluminescence measurements

The time-course bioluminescence results were obtained as reported previously (Isojima *et al*. 2009). PMT measurements with a high-sensitivity bioluminescence detection system (LM-2400; Hamamatsu Photonics, Hamamatsu, Japan) were started immediately after the start of PT slice culture at the time of light-off (ZT12 for neonatal PT slices, ZT8 or 16 for adult short- or long-day slices). For the data analysis in Fig. 3B,D, raw photon counts were plotted. For the data analysis in Fig. 3C,E and S3 in Supporting Information, photon counts of the PT slice from heterozygous *TSH*β^*Luc*^ mice were calculated by subtracting background photon counts of PT slice from wild-type mice. The resultant values were further normalized by photon counts at ZT4.75, which is 12.75 h after the start of the culture in adult long-day slices (Fig. 3C), or at ZT23, which is 11 h after the start of the culture in neonatal PT slices (Fig. 3E), which were the median time for the lowest bioluminescence values from all vehicle-treated samples in adult and neonatal slices, respectively (Fig. S4 in Supporting Information). The resultant values were normalized by photon counts at ZT13.45, which is just before glutamine and glutamic acid treatment in neonatal PT slices (Fig. S3 in Supporting Information).

### Bioluminescence imaging

Samples were prepared as described above. Sealed 35-mm culture dishes were placed on the stage of a macrozoom microscope (MVX10; Olympus, Tokyo, Japan) in a dark hood. The culture dishes were kept at approximately 37 °C in a heated chamber (Tokai Hit, Shizuoka, Japan) on the microscope stage. Bioluminescence was imaged using a 1.6 × Plan Apochromat objective (NA 0.24; Olympus) with 6.3 × zoom and transmitted to an electron-multiplying charge-coupled device (EM-CCD) camera cooled to −80 °C (ImagEM; Hamamatsu Photonics). The dimension of an image is 512 × 512 pixels, and each pixel corresponds to the size of 1.587 × 1.587 μm. Exposure time was 29 min with EM gain of 200 × for snapshot images, and exposure time was 14 min with EM gain of 300 × for time-lapse images. Images were transferred at 690 KHz to minimize readout noise, and analyzed using MetaMorph software (Molecular device, Sunnyvale, CA, USA). Single-cell tracking was performed as previously described (Ukai *et al*. 2007).

### Mass spectrometry

The YALSQDVCTYR peptide, which was the unique sequence for TSHβ, was synthesized on a peptide synthesizer (Syro Wave; Biotage, Uppsala, Sweden) using Fmoc solid-phase chemistry. The peptide was dimethyl-labeled with formaldehyde (CD_2_O) and desalted by C18 StageTips according to previously described method (Boersema *et al*. 2009). The dimethyl labeling of the peptide was checked by liquid chromatography coupled with tandem mass spectrometry (LC-MS/MS) with an LTQ orbitrap velos mass spectrometer (Thermo scientific, Waltham, MA, USA) coupled to a nano-Advance UHPLC system (Bruker Daltonics, Leipzig, Germany).

The culture medium of the PT slices was collected and temporarily stocked at −80 °C for further use. Proteolytic digestion was performed using a phase-transfer surfactant protocol (Masuda *et al*. 2008; Narumi *et al*. 2012). The resultant digest was dimethyl-labeled with formaldehyde (CH_2_O).

For quantification by mass spectrometry, a triple quadrupole mass spectrometer (TSQ Vantage EMR; Thermo scientific) was used to design a selected reaction monitoring (SRM) method. LC-SRM/MS was performed by a TSQ Vantage EMR mass spectrometer equipped with a nanoLC interface (AMR), a nano-Advance UHPLC system (Bruker Daltonics) and an HTC-PAL autosampler (CTC Analytics, Basingstoke, UK). The parameters of the mass spectrometer were set as follows: 0.002 m/z scan width, 40 msec scan time, 0.7 fwhm Q1 and Q3 resolution and 1.8 mTorr gas pressure.

For data analysis of the peak area in the chromatogram of each SRM transition, the peak area was extracted by using Pinpoint software (Thermo scientific). To confirm whether the peaks of SRM chromatogram were derived from the endogenous YALSQDVCTYR peptide, we checked that ratios among the peak areas of 6 SRM transitions were comparable with those of the standard YALSQDVCTYR peptide. Three of six SRM transitions, which had a lower variability in the ratio between the 18 runs of LC-SRM/MS analysis, were used for the quantification of the endogenous YALSQDVCTYR peptide. Chromatograms of SRM transition are shown in Fig. S5 in Supporting Information. Interassay coefficient of variation was 7.9%. The lower limit of sensitivity was 13.27 attomole.

### Statistical analyses

All data are shown as means ± SEM. Comparison of two groups was made using two-sample *t*-test. A value of *P* < 0.05 was considered as statistically significant. The analyses were performed by Microsoft Excel (Microsoft, Redmond, WA, USA) or R software (see The Foundation for Statistical Computing, http://www.R-project.org).

## Supporting Information

**Figure S1** (A) Time-lapse images of neonatal PT slice from heterozygous *TSH*β^*Luc*^ mice kept under L12:D12 conditions. Scale bar: 100 μm. (see also Video S1 in Supporting Information) (B) Representative bioluminescence patterns in individual neonatal PT cells in Fig. S1A. Scale bar: 100 μm.

**Figure S2** Confirmation of expression level changes of *TSH*β, *Eya3*, and *Cga* in the cultured PT by qPCR analysis.

**Figure S3** Real-time monitoring of the luciferase reporter activities in neonatal PT slices with glutamine or glutamic acid treatment.

**Figure S4** Histograms of the bottom time of bioluminescence of adult long-day PT samples (*n* = 19, upper) and neonatal PT samples (*n* = 157, bottom) that were treated with vehicle.

**Figure S5** The chromatograms of each selected reaction monitoring (SRM) transition.

**Video S1** Real-time imaging of bioluminescence in a neonatal PT slice for 4 days.
