# Supplementary figures and images for: Establishment of TSH β real-time monitoring system in mammalian photoperiodism
Source: Genes Cells. 2013 Jun 12;18(7):575–88. doi: 10.1111/gtc.12063 (PMC3738941; doi:10.1111/gtc.12063)

Figure S1

A

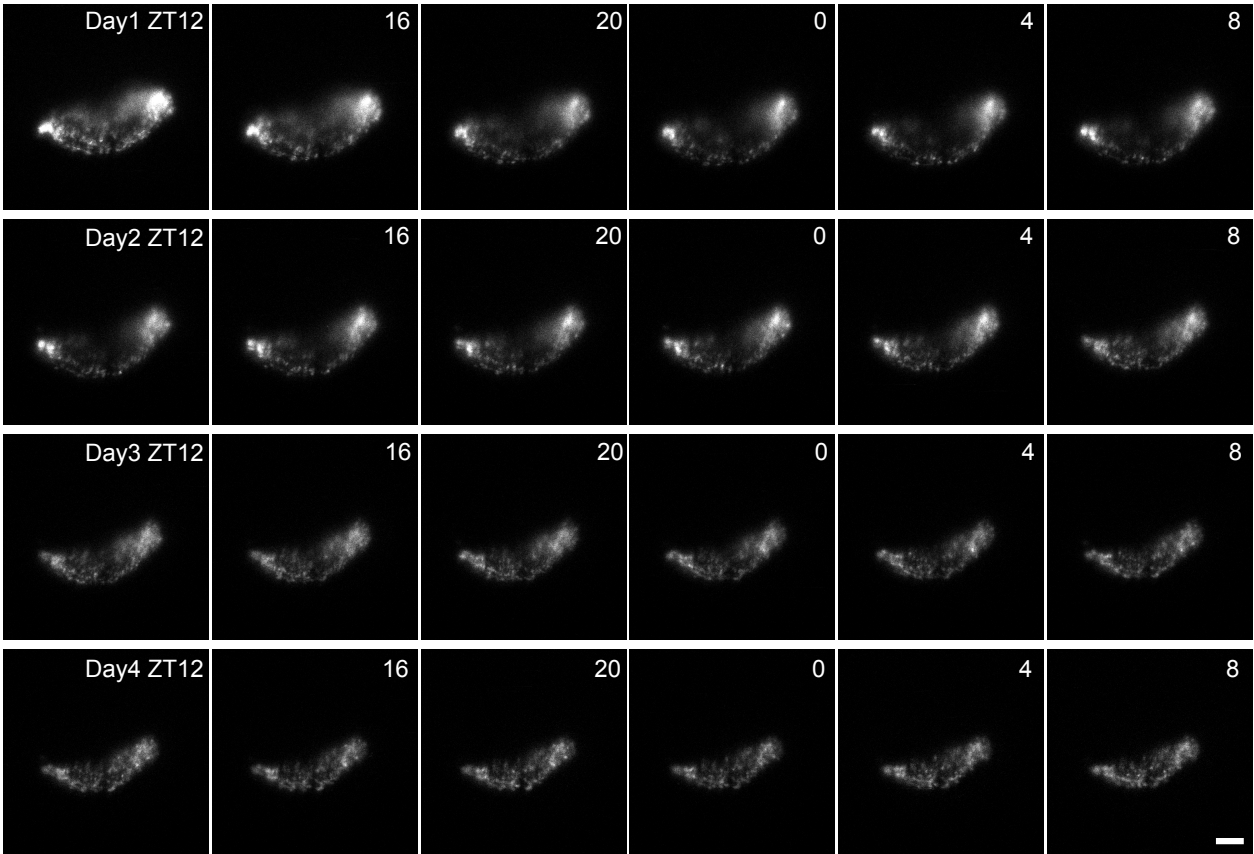

B

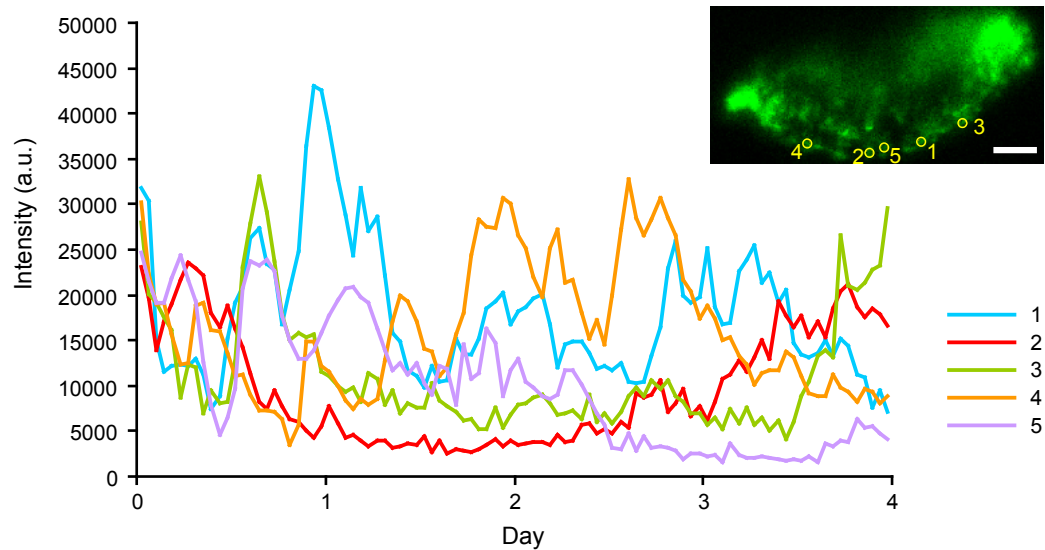

Supplement: Supplementary file 1 [file gtc0018-0575-SD1.pdf]

Figure S2

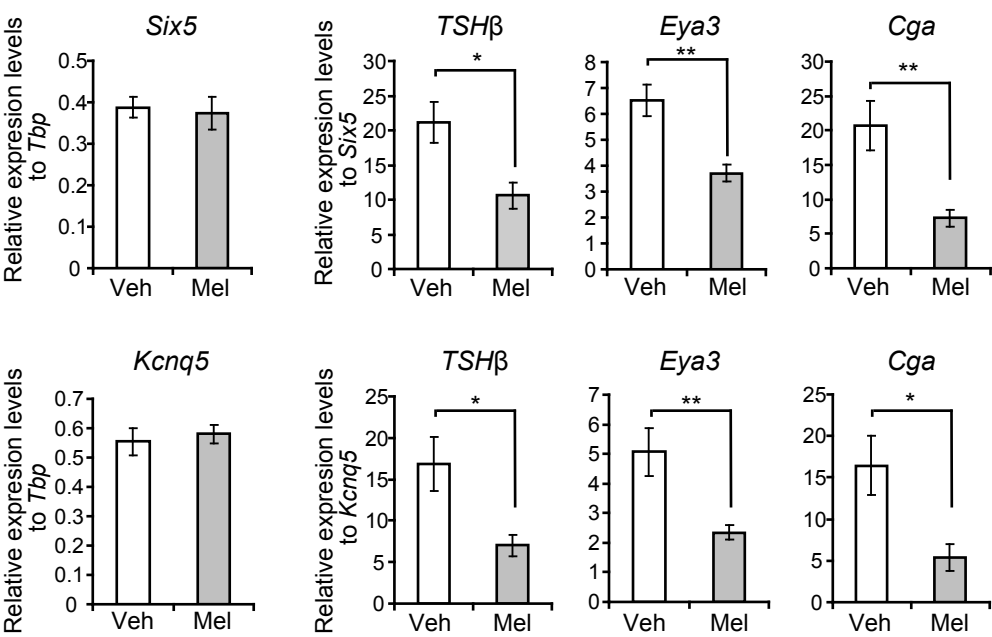

Supplement: Supplementary file 2 [file gtc0018-0575-SD2.pdf]

Figure S3

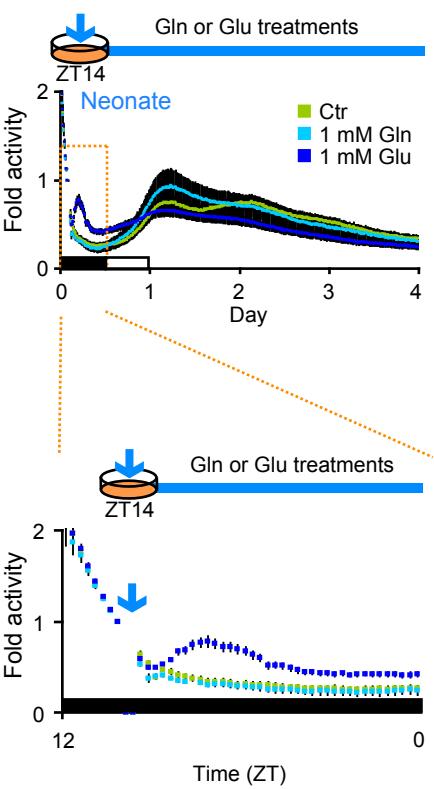

Supplement: Supplementary file 3 [file gtc0018-0575-SD3.pdf]

Figure S4

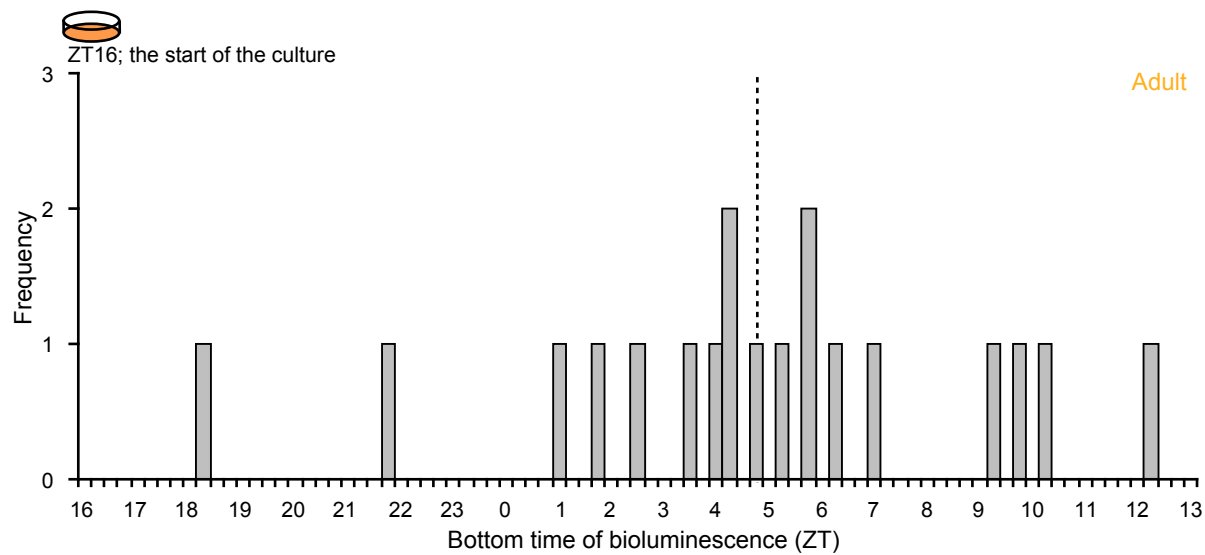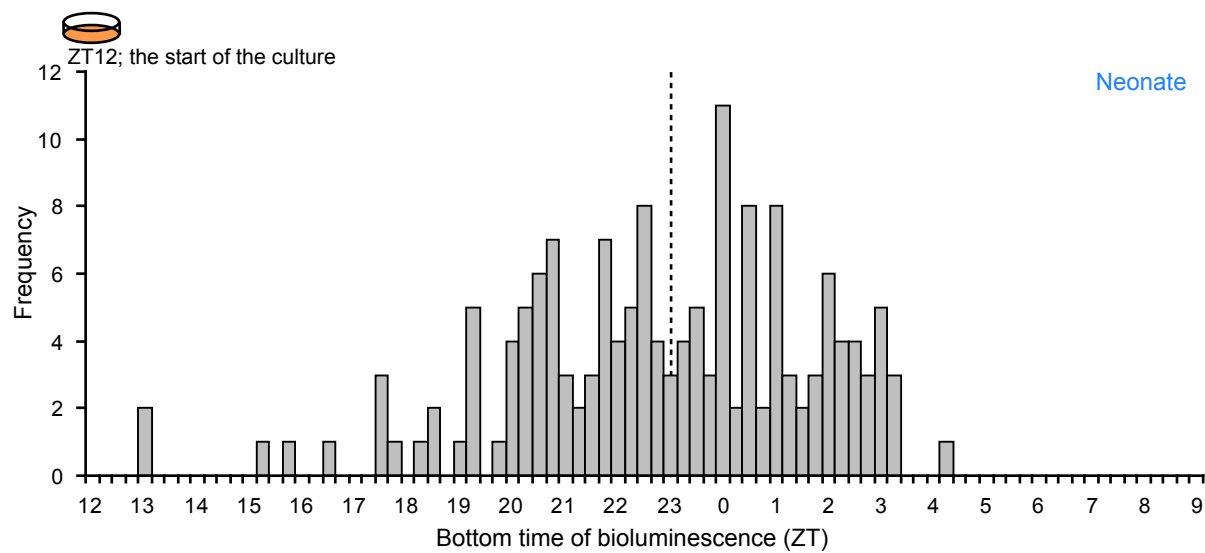

Supplement: Supplementary file 4 [file gtc0018-0575-SD4.pdf]
