# Supplementary material for: Establishment of TSH β real-time monitoring system in mammalian photoperiodism
Source: Genes Cells. 2013 Jun 12;18(7):575–88. doi: 10.1111/gtc.12063 (PMC3738941; doi:10.1111/gtc.12063)

Figure S5

A Workflow of the quantitation of TSHβ by LC-SRM/MS

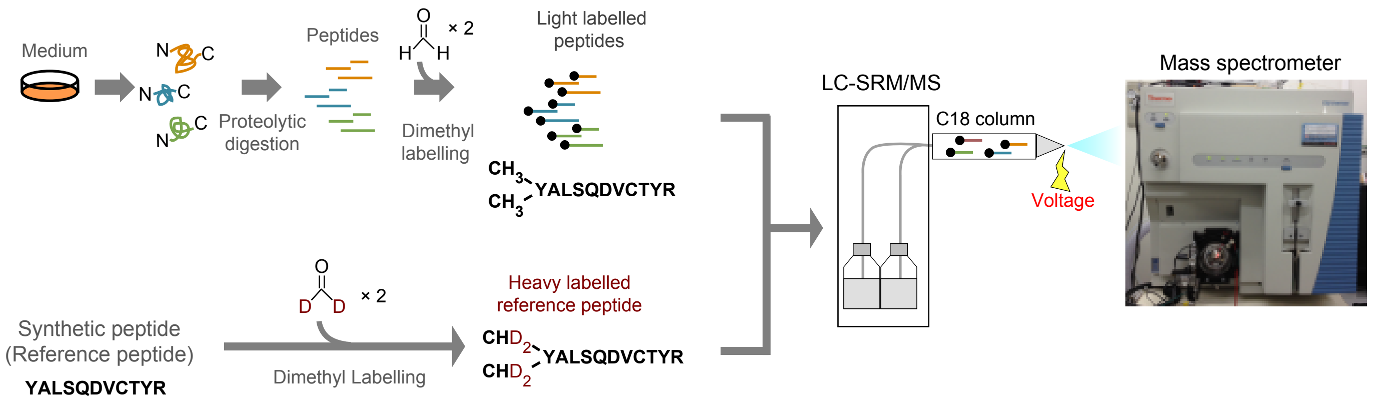

B

```
         10         20         30         40
MSAAVLLSVL FALACGQAAS FCIPTEYTMY VDRRECAYCL

         50         60         70         80
TINTTICAGY CMTRDINGKL FLPK[YALSQD VCTYR]DFIYR

         90        100        110        120
TVEIPGCPHH VTPYFSFPVA ISCKCGKCNT DNSDCIHEAV

        130
RTNYCTKPQS FYLGGFSV
```

C

Endogenous peptide

m/z (Precursor)	m/z (Product)	CE (V)	Ion type	Peptide sequence
702335	69833	35	y5	VCTYR
	813357	29	y6	DVCTYR
	941.415	28	y7	QDVCTYR
	1028.447	29	y8 †	SQDVCTYR
	1141531	28	y9 †	LSQDVCTYR
	1212568	28	y10†	ALSQDVCTYR

Reference peptide

m/z (Precursor)	m/z (Product)	CE (V)	Ion type	Peptide sequence
704348	69833	35	y5	VCTYR
	813357	29	y6	DVCTYR
	941415	28	y7	QDVCTYR
	1028447	29	y8 †	SQDVCTYR
	1141531	28	y9 †	LSQDVCTYR
	1212568	28	y10†	ALSQDVCTYR

D

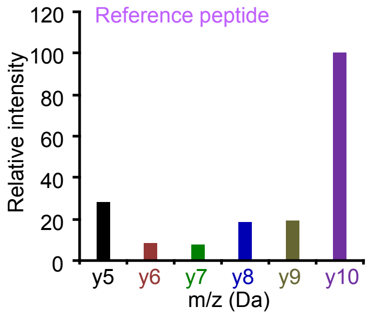

E

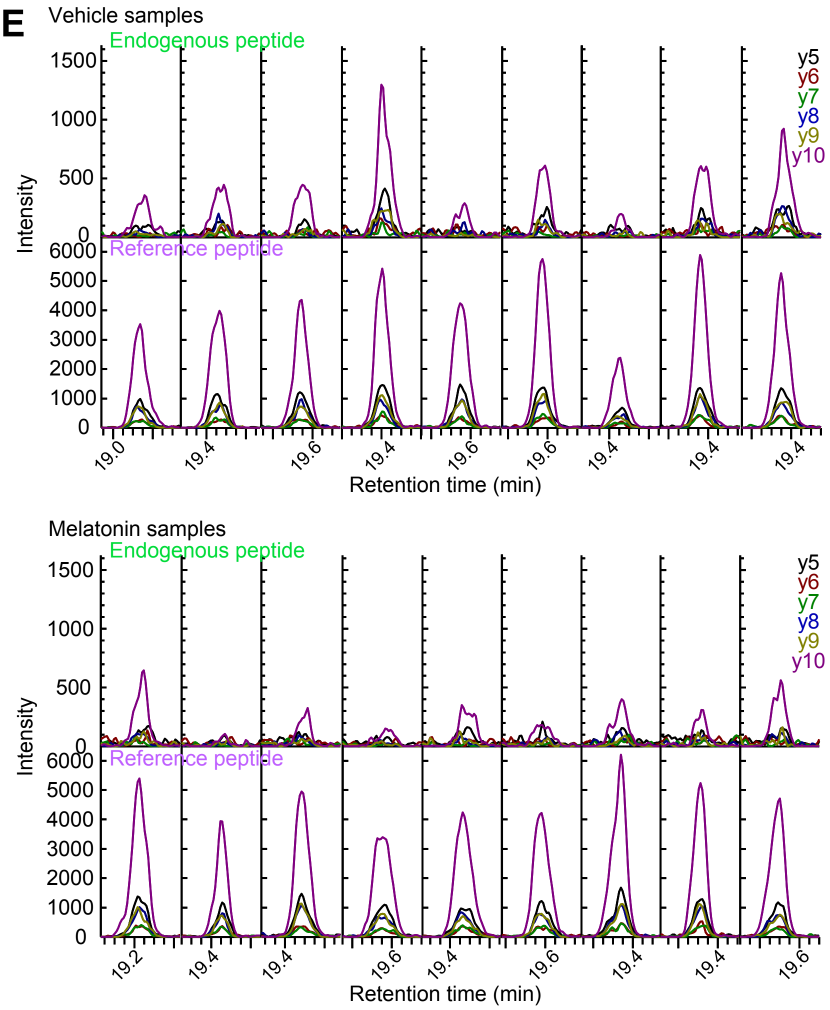

Supplement: Supplementary file 5 [file gtc0018-0575-SD5.pdf]
