# Supplementary material for: Establishment of TSH β real-time monitoring system in mammalian photoperiodism
Source: Genes Cells. 2013 Jun 12;18(7):575–88. doi: 10.1111/gtc.12063 (PMC3738941; doi:10.1111/gtc.12063)
Supplement: Supplementary file 11 [file gtc0018-0575-SD11.docx]

# Supporting Information

**Figure S1** (A) Time-lapse images of neonatal PT slice from heterozygous *TSH*β*^Luc^* mice kept under L12:D12 conditions. Scale bar: 100 μm. (see also **Video S1 in Supporting Information**) (B) Representative bioluminescence patterns in individual neonatal PT cells in **Fig. S1A**. Scale bar: 100 μm.

**Figure S2** Confirmation of expression level changes of *TSH*β, *Eya3*, and *Cga* in the cultured PT by qPCR analysis. Neonatal PT slices were treated with vehicle (Veh) or 10 nM melatonin (Mel). Expression levels of *TSH*β, *Eya3*, and *Cga* were normalized by the values of *Six5* and *Kcnq5,* which were specifically expressed in the PT. *, P < 0.05; **, P < 0.01, (two-sample *t* test, *n* = 9, average ± SEM). *Six5* normalized data are same data sets as in **Fig. 3F**.

**Figure S3** Real-time monitoring of the luciferase reporter activities in neonatal PT slices with glutamine or glutamic acid treatment. 1 mM glutamine (Gln) or 1 mM glutamic acid (Glu) containing medium and control medium that does not contain glutamine and glutamic acid (Ctr) was replaced 2 hr after glutamine and glutamic acid starvation from the start of the culture. Lower panel shows data that are 12 hr after the start of the culture in upper panel. White and black bars on the graphs indicate the subjective day and night on the following days after sampling, respectively. Normalized luciferase reporter activities of neonatal PT slices from heterozygous *TSH*β*^Luc^* mice kept under L12:D12 conditions are shown. (*n* = 5, average ± SEM).

**Figure S4** Histograms of the bottom time of bioluminescence of adult long-day PT samples (*n* = 19, upper) and neonatal PT samples (*n* = 157, bottom) that were treated with vehicle. The median time (ZT4.75 in adult long-day, ZT23 in neonate) was indicated as dash lines. ZT16 in upper panel and ZT12 in lower panel mean the start time of the culture.

**Figure S5** The chromatograms of each selected reaction monitoring (SRM) transition. (A) Experimental schema. The culture medium of the PT slices was collected and digested. The resultant digest was dimethyl labelled with formaldehyde (CH_2_O). Synthetic peptide for reference was dimethyl labelled with formaldehyde (CD_2_O). Each peptide was desalted by C18 StageTips and quantified by mass spectrometry. See Experimental procedures. (B) Amino acid sequence of TSHβ. Box indicates reference peptide sequence. (C) SRM transition parameters. CE means collision energy. †; The SRM transitions were used for quantification. (D) Relative intensity of each SRM transitions of reference peptides. (E) Chromatograms of endogenous peptides from the medium (each upper column) and synthetic peptides (each bottom column) are shown. The x-axis scale is 0.05 min per division.

**Video S1** Real-time imaging of bioluminescence in a neonatal PT slice for 4 days.
